# Inflammation-Driven Plaque Erosion in Atherosclerosis: A Focus on Complement System Pathways

**DOI:** 10.1007/s11883-025-01279-x

**Published:** 2025-03-22

**Authors:** Davide Ramoni, Federico Carbone, Simon Kraler, Davide Di Vece, Fabrizio Montecucco, Luca Liberale

**Affiliations:** 1 Department of Internal Medicine, University of Genoa, 6 Viale Benedetto XV, 16132 Genoa, Italy; 2 IRCCS Ospedale Policlinico San Martino Genoa, Italian Cardiovascular Network, Largo Rosanna Benzi 10, 16132 Genoa, Italy; 3 Center for Molecular Cardiology, University of Zurich, Schlieren, Switzerland; 4 Department of Cardiology and Internal Medicine, Cantonal Hospital Baden, Baden, Switzerland; 5 Department of Internal Medicine B, University Medicine Greifswald, Greifswald, Germany

**Keywords:** Anti-Complement Therapies, Atherosclerosis, Cardiovascular diseases, Complement system, Inflammaging, Plaque erosion

## Abstract

**Purpose of Review:**

Complement system activation is implicated in various stages of atherogenesis, from fatty streak formation to plaque destabilization and thrombus formation, with its dreadful clinical sequelae such as myocardial infarction, stroke and premature death. In this review, we consider these issues and explore recent studies on complement activation in atherosclerotic plaque initiation and progression.

**Recent Findings:**

Complement pathways impact plaque stability and healing through the modulation of inflammatory processes. Recent studies indicate that complement components, notably C3 and C5b-9, accelerate atherosclerosis progression through their interactions with endothelial cells, smooth muscle cells, and immune cells. Nonetheless, the beneficial versus deleterious effects of complement activation at different stages of atherogenesis remains a matter of ongoing debates. Research also investigates therapies targeting the complement cascade to mitigate plaque erosion and rupture.

**Summary:**

This review explores the ongoing debates surrounding complement activation in atherogenesis. We bring forward controversial findings and therapeutic strategies aimed at modulating complement cascade activation with the ultimate goal to reduce the burden of atherosclerotic cardiovascular disease.\

## Introduction

Atherogenesis is a multi-step process driven by an intricate interplay of endothelial dysfunction, lipid homing and immune cell infiltration, resulting in the build-up of atherosclerotic plaques, with plaque rupture and erosion causing the vast majority of acute ischaemic events [1]. We now recognize that atherosclerosis is not solely driven by traditional factors such as elevated cholesterol, hypertension, and smoking, but also by non-traditional factors, including low-grade inflammation [2]. Indeed, chronic inflammation plays a central role in the initiation and progression of atherosclerosis [3]. Inflammatory responses, triggered by various stimuli, lead to the activation of immune cells and the production of pro-inflammatory cytokines that may accelerate plaque formation.

With increasing age, low-grade and sustained chronic inflammation (hereafter referred to as "*inflammaging*") exacerbates, markedly contributing to cardiovascular disease (CVD) risk [4, 5]. In line with this observation, somatic mutations in stem cells —such as clonal hematopoiesis of indeterminate potential (CHIP)— link to an overactive immune response and inflammation, which further promote atherosclerosis in certain vascular beds [6]. Obesity, specifically the accumulation of epicardial and visceral fat, also contributes to systemic inflammation, creating a pro-inflammatory environment that accelerates vascular damage [7, 8]. The gut microbiome and environmental factors like air pollution also play a role in systemic inflammation, with microbial imbalances driving proinflammatory and -atherogenic pathways [9]. These non-traditional factors underscore the multifactorial nature of atherosclerosis, highlighting the importance of targeting inflammation and its related drivers in preventing CVD [3, 10].

The complement system, a critical part of the innate immune system, involves a variety of proteins that detect and eliminate pathogens and damaged cells. Once activated, it boosts phagocytosis, recruits immune cells, and forms complexes to destroy harmful cells. Tight regulation is essential, as unchecked activation can lead to inflammation and tissue injury. Complement activation is closely tied to inflammatory processes, enhancing immune responses through the release of cytokines and chemotactic factors. Beyond defense, it supports tissue homeostasis by clearing apoptotic cells. However, its dysregulation is implicated in autoimmune, neurodegenerative, and CVD.

While vital for immunity and tissue clearance, complement overactivation may contribute to CVD. Such dual action makes it a very interesting player, although difficult to target, in the process of atherosclerosis. Understanding its molecular mechanisms is crucial for developing targeted therapies aimed at reducing adverse cardiovascular events. Here, we will focus on the role of complement in plaque instability and erosion.

## Atherosclerotic Plaque Vulnerability: Rupture and Erosion Dynamics

Acute ischemic cardio- and cerebrovascular events are mostly sustained by a sudden alteration of atherosclerotic plaque homeostasis through plaque rupture (macrophage-driven process) or erosion (neutrophil-driven process) leading to arterial thrombus formation and disrupting blood flow [11]. One remarkable aspect of these events is that they frequently occur in plaques with less than 50% stenosis, meaning that the artery is not significantly narrowed by the plaque itself [12]. This challenges the conventional belief that only severely obstructive plaques are dangerous, emphasizing the importance of plaque vulnerability rather than merely the degree of narrowing [13].

Plaque rupture stands out as the predominant mechanism behind coronary thrombosis, it sustained by the structural failure of the fibrous cap overlying the plaque necrotic core leading to its exposure to the blood stream. An extensive review of 22 autopsy studies involving nearly 2,000 coronary arteries found that plaque rupture was responsible for most coronary thrombosis cases (79%), regardless of how the condition manifested clinically. The reviewed autopsy studies showed similar rates of plaque rupture across continents: 72% in Europe, 68% in the United States, and 81% in Asia [14]. Results similar to those showed by in-vivo studies using intracoronary imaging [15]. This uniformity across diverse populations suggests that the underlying mechanisms driving plaque rupture could be universal, highlights the fundamental biological processes at work in atherosclerosis. Increased age and male sex are known risk factors for plaque destabilization through plaque rupture [16]. The risk of plaque rupture is closely tied to the structure of the fibrous cap covering the plaque. Thin-cap fibroatheromas (TCFA) are particularly prone to rupture due to their fragile structure. Specifically, the thin cap is unable to withstand the mechanical stress exerted by blood flow, making it more likely to rupture under conditions of high shear stress or inflammation. Studies using optical coherence tomography (OCT) have confirmed that in patients presenting with myocardial infarction, plaque rupture is more common (73%), while plaque erosion accounts for about 23% of cases. Moreover, 83% of ruptured plaques were identified as TCFA, underscoring the importance of fibrous cap integrity in determining plaque stability [17].

**Fig. 1:**
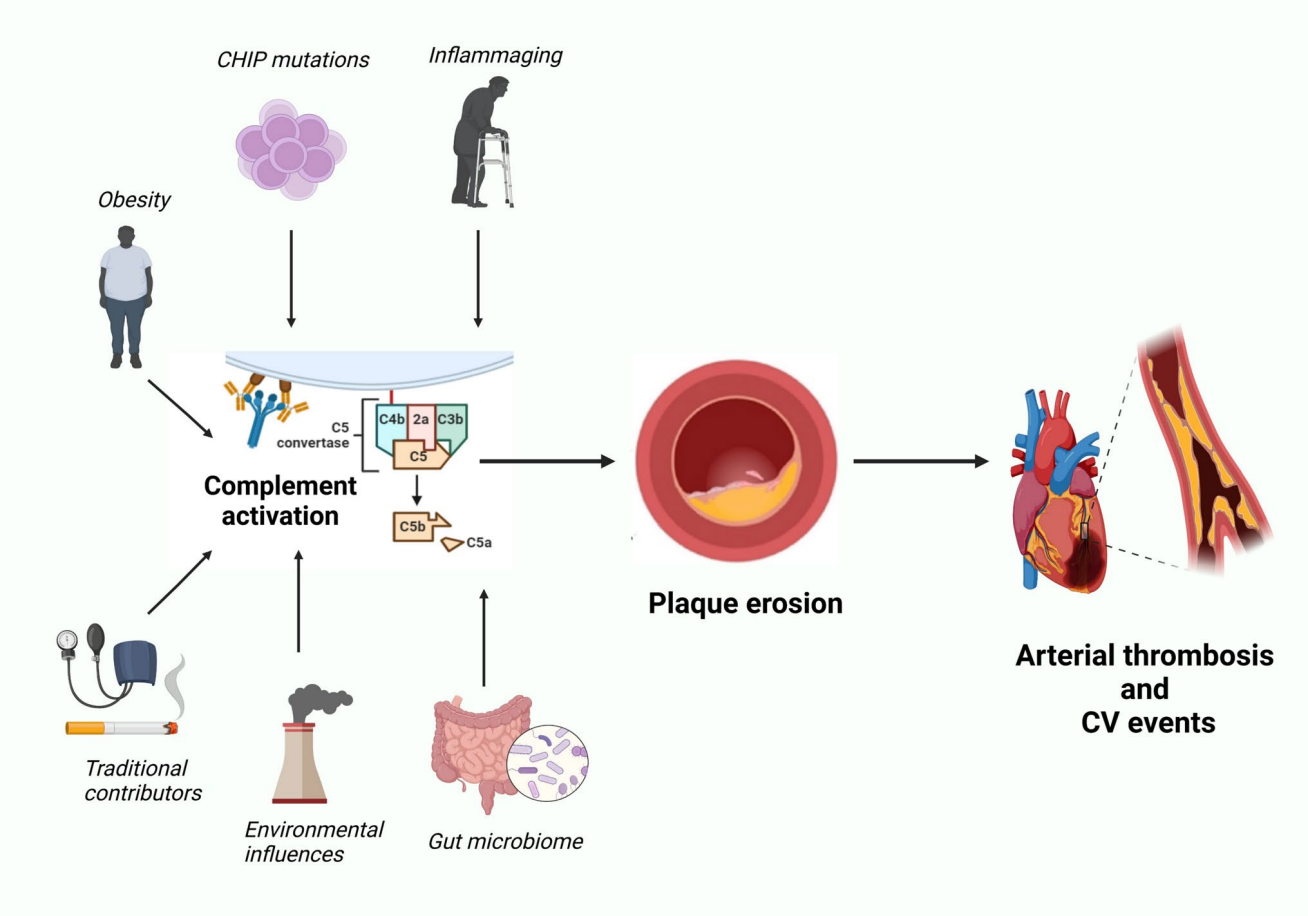
“Progression from atherosclerotic risk factors to plaque erosion and thrombotic events via complement activation". This network diagram illustrates the pathway leading from risk factors to acute vascular events. On the left, risk factors for atherosclerotic plaque formation—both established and emerging—are shown, each contributing to the activation of the complement system. The complement activation further promotes plaque erosion, characterized by the loss of the protective endothelial layer and exposure of the underlying plaque to circulating blood. This process increases vulnerability to thrombus formation, as the eroded plaque becomes a site for platelet adhesion and aggregation. The resulting thrombosis can then trigger acute cardio- and cerebrovascular events. Created in BioRender. Montecucco, F. (2024)

Though plaque rupture is the most common cause of coronary thrombosis, plaque erosion is emerging as another critical mechanism, which is increasing in its prevalence due to population ageing and improved secondary prevention measures [15]. Unlike rupture, erosion involves the loss of the endothelial lining over the plaque (so-called endothelial denudation), leaving the underlying vascular smooth muscle cells (SMCs) and proteoglycans exposed. Such a process triggers thrombus formation while leaving the fibrous cap structure intact. While erosion and rupture are distinct processes, they share a common underlying feature: the weakening of the protective barrier between the plaque’s contents and the bloodstream. Notably, plaque erosion seems to affect a different demographic being more frequently reported in young patients, women (although this finding is still debatable), and those without the classic risk factors for atherosclerosis, such as dyslipidemia and smoking habits [18]. These findings suggest that the mechanisms behind plaque erosion may differ from those of rupture, potentially involving more subtle forms of endothelial dysfunction or local inflammatory processes. In clinical practice, this means that patients who might not exhibit traditional risk markers could still be vulnerable to plaque erosion and the subsequent development of acute coronary syndromes (Fig. 1).

While complement activation has long been associated with inflammation and tissue damage, its specific role in plaque erosion, as distinct from rupture, is gaining increasing attention.

## Complement System Activation and its Role in Plaque Erosion

The role of the complement system in atherosclerosis, particularly in plaque erosion, is complex and multifaceted [19]. As an essential part of both innate and adaptive immunity, complement activation can either modulate inflammation or exacerbate vascular damage depending on the specific conditions and the stage of plaque development. The complement system can be activated via three distinct pathways: the classical, lectin, and alternative pathways. Levels of complement components such as C1q, C3, and C5a are notably elevated in atherosclerotic plaques compared to normal arterial tissue. Moreover, the presence of activated complement components in atherosclerotic plaques has been associated with increased plaque instability [20–22].

One of the most compelling aspects of complement's involvement in plaque erosion is its ability to serve as both a protector and a potential harm to vascular integrity, contingent on the context and extent of activation. At low activation levels, complement plays a crucial role in clearing apoptotic cells and preventing excessive inflammation, a vital process during the early stages of atherosclerosis [23]. However, as activation becomes excessive, it can lead to detrimental effects, such as the detachment of endothelial cells, which exposes the underlying pro-thrombotic layers [18].

Despite significant advancements in our understanding of the complement system, several controversies and unresolved questions persist regarding its role in plaque erosion. Central to these debates is whether complement activation acts primarily as a protective mechanism in early atherosclerosis or becomes a pathogenic driver in advanced disease stages.

Primarily triggered by immune complexes, the classical pathway of the complement system has been associated with a protective role in atherosclerosis. C1q, a key component of this pathway, facilitates the clearance of apoptotic cells and debris, thereby preventing excessive inflammation [20]. Several studies have demonstrated that C1q is expressed by dendritic cells, macrophages, and foam cells within the atherosclerotic plaque, where it contributes to plaque stability by limiting the accumulation of necrotic core material [24, 25]. However, as atherosclerosis progresses, even the classical pathway can become overactivated, leading to chronic inflammation and plaque destabilization [26].

The lectin pathway, activated by pathogen-associated molecular patterns (PAMPs), has recently gained attention for its role in plaque vulnerability. Serum levels of ficolin-2, a critical protein in the lectin pathway, have been shown to predict acute coronary syndromes in patients with severe carotid stenosis [27]. Moreover, studies have linked the activation of the lectin pathway to an increased risk of plaque erosion, characterized by the loss of the endothelial lining and exposure of the underlying SMCs-rich intima [28].

The alternative pathway, which remains continuously active at low levels, can be amplified by inflammatory stimuli such as enzymatically modified LDL, oxLDL, and cholesterol crystals (CCs) [29, 30]. Such mediators, prevalent in atherosclerotic plaques, activate both the classical and alternative complement pathways by binding to C-reactive protein (CRP), further intensifying the inflammatory cascade [31, 32]. The alternative pathway is more pro-inflammatory than the classical pathway and is strongly linked to plaque progression and instability [33].

Cleavage of C3 and C5 are central steps in the complement cascade, profoundly impacting plaque development and stability. C3 activation is particularly crucial in the early stages of atherosclerosis, as it facilitates the clearance of apoptotic cells and debris, thereby reducing the likelihood of necrotic core formation. However, once a critical threshold of inflammation is reached, C3 cleavage can propagate a detrimental inflammatory response [34]. Studies have shown that high levels of C3 correlate with an increased risk of CV diseases, suggesting that systemic complement activation reflects local plaque activity [21].

All complement pathways converge at the activation of C3, which results in further cleavage to C3a and C3b [33]. This is followed by the activation of C5 and the formation of the membrane attack complex (MAC or C5b-9), which plays a pivotal role in destabilizing atherosclerotic plaques, particularly through its effects on the endothelial layer [35, 36]. While C5b-9 is traditionally known for its cytolytic ability against pathogens, its sublytic effects on endothelial cells are critical for promoting plaque erosion. Sublytic concentrations of C5b-9 can induce endothelial dysfunction without immediate lysis, resulting in increased permeability, enhanced expression of adhesion molecules, and a heightened inflammatory state [37, 38].

Endothelial dysfunction caused by C5b-9 has been linked to the upregulation of pro-inflammatory molecules such as TNF-α, which further exacerbates local inflammation [39, 40]. This inflammatory environment can degrade the extracellular matrix and weaken the attachment of endothelial cells to the underlying intima, leading to their detachment and exposing the SMCs beneath, the hallmark of plaque erosion. Sublytic concentrations of C5b-9 on SMCs promote their proliferation and migration, critical processes in the early stages of atherosclerotic plaque development [41]. Complement activation, particularly through C5a and the MAC, can induce the release of monocyte chemoattractant protein-1 (MCP-1) from SMCs, attracting monocytes to the site of erosion [42]. These monocytes differentiate into macrophages and foam cells, further fueling the inflammatory response and leading to thrombus formation [43].

Furthermore, complement activation in SMCs can inhibit apoptosis, a process that would typically limit SMCs proliferation and migration [44]. By preventing apoptosis, sublytic C5b-9 allows for continued SMCs activity, contributing to plaque instability and increasing the risk of further erosion. Among the complement components, C5a is a potent pro-inflammatory molecule. Upon activation, C5 is cleaved into C5a and C5b, with C5a serving as a chemoattractant recruiting neutrophils and monocytes to the site of complement activation. These immune cells release proteolytic enzymes that degrade the extracellular matrix, promoting the detachment of endothelial cells and initiating erosion. In this scenario, the C5a-C5aR axis has been identified as a major driver of atherosclerosis progression and destabilization. C5a binds to its receptor, C5aR, on endothelial and immune cells, amplifying the local inflammatory response [45]. This interaction leads to the upregulation of adhesion molecules such as VCAM-1 and ICAM-1 on the endothelial surface, facilitating the recruitment of inflammatory cells to the plaque. The resulting inflammatory milieu weakens the endothelial layer and increases the likelihood of its detachment. Additionally, C5a can induce endothelial cell apoptosis, further compromising the integrity of the endothelial lining. In animal models of atherosclerosis, inhibiting C5aR has been shown to reduce plaque size and prevent erosion, underscoring C5a's critical role in the erosion process [46].

Finally, the role of CCs in atherosclerotic plaque is discussed. CCs are abundant in advanced atherosclerotic plaques, particularly in those prone to rupture [47]. Macrophages that internalize CCs through their scavenger receptors undergo a pro-inflammatory activation, releasing cytokines and reactive oxygen species and serve as potent triggers for complement activation, that further drive inflammation. These crystals can activate the complement system through the alternative pathway, leading to the deposition of complement components [48]. The role in plaque erosion is, however, more debated. Studies have shown that CCs not only promote complement activation but also stimulate the release of procoagulant molecules, linking complement activity with thrombus formation following plaque erosion. The ability of CCs to activate complement has been demonstrated in both in vitro and in vivo studies, where they have been shown to leads to the recruitment of neutrophils and induce neutrophil extracellular traps (NETs), associated with plaque destabilization and erosion [49]. NETs, composed of DNA and proteolytic enzymes, further degrade the extracellular matrix and promote thrombosis, establishing a vicious cycle of inflammation and vascular damage. NETs are also known to activate platelets, creating a pro-thrombotic environment conducive to clot formation following erosion [50].

With complement activation suggested to be protective at early stages and deleterious during the latest phases of atherosclerosis, its role as a therapeutic target is debated. As already suggested for other anti-inflammatory treatments [51], the optimal timing and dosage for anti-complement therapies may be the pathway for successfully implementing such strategy in the clinical context. Also, given the role of complement in plaque erosion and taking into consideration potential sex-differences in this phenomenon, complement-targeted therapies may need different tailoring according to the sex [14].

## Anti-Complement Therapies to Mitigate Cardiovascular Risk

Experimental anti-complement therapies targeting specific components of the complement cascade have shown promise in preclinical models; however, translating these findings into clinical practice has proven challenging. The complexity of the complement system, with its extensive involvement in various physiological processes, poses a significant hurdle. While inhibiting complement activation may mitigate plaque erosion and rupture, it could also impair host defense mechanisms, leading to increased susceptibility to infections.

Several complement inhibitors are currently available although the evidence for their applicability to reduce CV burden remains very primitive and often limited to preclinical models (Table 1). Eculizumab, a monoclonal antibody that blocks C5 activation, has demonstrated potential in reducing complement-mediated damage in conditions like paroxysmal nocturnal hemoglobinuria (PNH) [52]. However, its application in atherosclerosis remains limited by safety and efficacy concerns [53]. Similarly, CD59 serves as a key regulator of MAC assembly, with its absence leading to more severe plaque formation and increased myocardial infarction incidence in experimental models. Studies have shown that complement inhibition using a neutralizing anti-mouse C5 antibody attenuates atherosclerosis, indicating the importance of the terminal complement pathway in disease progression [54].

**Table 1:** Currently available specific anti-complement therapies and their potential in plaque erosion

Therapy	Target	Mechanism of Action	Research stage	Relevance to Atherosclerosis
Eculizumab [53, 64]	C5	Inhibits C5 activation, preventing formation of C5a (pro-inflammatory mediator) and C5b (MAC component)	Approved for PNH; limited trials in CV disease	Reduces inflammation; may decrease plaque vulnerability by preventing MAC-driven endothelial damage
Pexelizumab [55, 56, 65,57,59]	C5	Monoclonal antibody inhibiting C5 activation, aiming to reduce inflammation and thrombosis	Tested in phase III trials (e.g., PRIMO-CABG II; APEX AMI) but results inconclusive in CV	Limited efficacy in reducing CV events; insights suggest challenges in targeting C5 in atherosclerosis
C1-INH [58, 66]	C1 esterase	Inhibits classical pathway, reducing inflammation and immune complex activation	Investigated in myocardial infarction patients; approved for angioedema	Shows potential in reducing ischemia–reperfusion injury; may stabilize plaques by minimizing inflammatory cascades
Compstatin (CP40) [53, 61]	C3	Prevents C3 activation, reducing downstream inflammation and immune cell recruitment	Preclinical stages for CV applications	Blocks upstream complement pathway, potentially reducing generation of PTF1.2 and monocyte TF expression mediated by CCs
Coversin (Nomacopan) [60, 67]	C5	Dual inhibitor targeting C5 and leukotriene B4 to reduce inflammation and vascular damage	Treatment of Bullous Pemphigoid and PNH	Potential to prevent C5 activation in atherosclerotic plaques, limiting endothelial damage and thrombosis
Ravulizumab [68]	C5	Long-acting C5 inhibitor reducing MAC formation and inflammatory effects	Approved for PNH, aHUS and generalised myasthenia gravis	May provide extended protection against MAC-driven plaque erosion and rupture
Sutimlimab [69]	C1s	Inhibits C1s, targeting classical pathway; reduces inflammation linked to immune complexes	Approved for cold agglutinin disease	Potential to reduce inflammation-driven plaque instability through upstream inhibition
PMX53 [53, 62]	C5aR1	Blocks C5a receptor (C5aR1), preventing pro-inflammatory and chemotactic effects of C5a	Preclinical studies for CVD; limited trials	Reduces recruitment of inflammatory cells to plaques, possibly preventing destabilization; blocked CCs-induced PTF1.2 and reduced TF monocytes
Vilobelimab [70]	C5a	Anti-C5a antibody that neutralizes C5a, limiting local inflammation and immune cell recruitment	FDA emergency use for severe COVID-19	Potential to mitigate inflammation in atherosclerosis; may stabilize plaques by preventing C5a-induced cell recruitment

atypical hemolytic uremic syndrome (aHUS); cardiovascular disease (CVD); cholesterol crystals (CCs); membrane attack complex (MAC); paroxysmal nocturnal hemoglobinuria (PNH); prothrombin fragment 1 + 2 (PTF 1.2); tissue factor (TF)

Pexelizumab, another monoclonal antibody targeting complement C5, initially showed promising results in early-phase II clinical trials, such as the COMMA [55] and CABG trials [56], demonstrating a 30% reduction in 30-day mortality among patients with acute myocardial infarction. However, larger trials like PRIMO-CABG II, which involved more than 4,000 patients combined, failed to replicate these findings, with no significant benefits observed in reducing mortality or myocardial injury [57]. One reason for this lack of success may be the persistent generation of the terminal complement complex sC5b-9, which Pexelizumab could not adequately control, despite its effectiveness in inhibiting C5a and IL-6 elevation.

Conversely, upstream inhibition of the complement system using C1 esterase inhibitor (C1-INH) has shown more promise. Studies involving patients undergoing emergency coronary artery bypass grafting due to acute ST-elevation myocardial infarction indicated that C1-INH reduced myocardial ischemia–reperfusion injury and improved myocardial contractility [58]. This suggests that targeting earlier components of the complement cascade may offer a more effective strategy for reducing inflammatory damage associated with acute myocardial infarction compared to C5-targeted therapies like Pexelizumab [59].

Coversin (Nomacopan) has been studied for conditions such as bullous pemphigoid and PNH as an anti-complement therapy targeting both C5 and leukotriene B4, aiming to reduce inflammation and prevent vascular damage [60]. Yet, to date no specific data on CV conditions is available. Inhibition of complement C3 by CP40 (compstatin) or C5aR1 by PMX53 has demonstrated promising experimental effects in blocking monocyte tissue factor expression, further supporting the potential of these therapeutic approaches [61, 62].

Additionally, monoclonal antibodies like Ravulizumab (a humanized anti-C5 antibody) and Sutimlimab (a humanized anti-C1s antibody) have gained attention for their roles in treating conditions associated with complement dysregulation. Ravulizumab has been approved for treating PNH and atypical hemolytic uremic syndrome (aHUS), while Sutimlimab has been approved for cold agglutinin disease. Furthermore, Vilobelimab, an anti-C5a antibody, received FDA emergency use approval for treating critically ill COVID-19 patients, a conditions associated with systemic endothelial dysfunction and suggesting its potential translatability to the cardiology arena [63]. Nevertheless, safety data for many of these treatments are still limited.

Despite these advancements, anti-complement therapies are not yet standard in clinical practice, and well-designed studies are urgently warranted to evaluate their efficacy in stabilizing atherosclerotic plaques, minimize side effects and preventing further cardiovascular events.

## Conclusions

Although the role of complement in atherosclerosis is experimentally well-established, significant challenges remain in translating this knowledge into effective therapies. Future research must deepen our knowledge into the precise mechanisms and timing through which complement activation contributes to plaque erosion. Further, we need to explore innovative strategies for targeting this pathways in atherosclerotic cardiovascular disease. Excessive complement activation, particularly through the terminal complement pathway and C5a, may play a crucial role in the development of plaque erosion. Indeed, while the deposition of sublytic C5b-9 on endothelial and SMCs promotes endothelial dysfunction and intimal exposure, C5a-driven inflammation accelerates arterial thrombosis. Even though plaque rupture remains the dominant cause of thrombosis, plaque erosion is increasingly recognized as a significant contributor to acute cardiovascular events, particularly in younger individuals, in which incident cardiovascular events are plateauing [71]. The interplay between CCs, NETs, and complement further amplifies these processes, creating a damaging cycle of endothelial injury, inflammation, and thrombosis that culminates, despite the often-silent nature of these processes, in sudden and catastrophic events. Addressing complement activation therapeutically may open new avenues for preventing plaque erosion and its devastating clinical consequences.

## Key References

- Libby P. Inflammation and the pathogenesis of atherosclerosis. Vascul Pharmacol. 2024 Mar;154:107,255. doi: 10.1016/j.vph.2023.107255. Epub 2023 Dec 28. PMID: 38,157,682.
  - Peter Libby's research establishes inflammation as central to atherosclerosis development and plaque instability, involving immune cells that migrate to arterial walls. Monocytes evolve into macrophages and foam cells, critical in plaque growth. Anti-inflammatory treatments like IL-1β inhibitors have shown promise in reducing CV events when combined with cholesterol-lowering therapy, suggesting a novel approach to residual risk management in atherosclerosis.

- Kiss MG, Binder CJ. The multifaceted impact of complement on atherosclerosis. Atherosclerosis. 2022 Jun;351:29–40. doi: 10.1016/j.atherosclerosis.2022.03.014. Epub 2022 Mar 17. PMID: 35,365,353.
  - The authors explore the complement roles in atherosclerosis, play both protective and harmful action. Complement can exacerbate inflammation, promote foam cell formation, and destabilize plaques, yet also help clear dead cells and pathogens from atherosclerotic lesions. Their findings suggest targeting specific pathways in the complement system could help manage inflammation and reduce CV risks associated with atherosclerosis.

- Smith PK, Shernan SK, Chen JC, Carrier M, Verrier ED, Adams PX, Todaro TG, Muhlbaier LH, Levy JH; PRIMO-CABG II Investigators. Effects of C5 complement inhibitor pexelizumab on outcome in high-risk coronary artery bypass grafting: combined results from the PRIMO-CABG I and II trials. J Thorac Cardiovasc Surg. 2011 Jul;142(1):89–98. doi: 10.1016/j.jtcvs.2010.08.035. Epub 2010 Sep 28. PMID: 20,880,552.
  - Larger trials using C5 complement inhibition pexelizumab failed to demonstrate reduction in the primary composite endpoint of death or MI at postoperative day 30 in CABG patients.

## Data Availability

No datasets were generated or analysed during the current study.
